# SNORA71B promotes breast cancer cells across blood–brain barrier by inducing epithelial-mesenchymal transition

**DOI:** 10.1007/s12282-020-01111-1

**Published:** 2020-05-23

**Authors:** Sijia Duan, Xuliang Luo, Huihui Zeng, Xiang Zhan, Chunlei Yuan

**Affiliations:** 1 Department of Breast Surgery, The Second Affiliated Hospital of Nanchang University, No.1 Mingde Road, Donghu District, Nanchang, 330006 Jiangxi Province China; 2 Medical College of Nanchang University, Nanchang, 330006 Jiangxi People’s Republic of China; 3 The People’s Hospital of Le ‘An County, Fuzhou, 344000 Jiangxi China

**Keywords:** SNORA71B, Brain metastases, Breast cancer, Epithelial-mesenchymal transition, Blood–brain barrier

## Abstract

**Background:**

Brain metastasis (BM) is a dreadful complication that significantly impacts the quality of life in breast cancer patients. A key process during brain metastasis is the migration of cancer cells across blood–brain barrier (BBB). However, the role of snoRNAs regulating BBB in BM is still unknown.

**Methods:**

Here SNORic and GEO databases were used to identify differentially expressed snoRNAs between brain metastatic and non-metastatic breast cancer (BC) tissues. The effects of SNORA71B on the capacities of proliferation, migration, invasion, epithelial-mesenchymal transition (EMT), and BBB invasion of BC cells were evaluated by CCK8, transwell, western blot, and BBB model, respectively.

**Results:**

SNORA71B was highly expressed in high BM BC tissues and cells compared to low BM BC controls. Survival analysis revealed high expression of SNORA71B was significantly associated with poor PPS and OS in breast cancer patients. ROC curve showed that SNORA71B might act as biomarker for breast cancer. Moreover, SNORA71B significantly promoted proliferation, migration, and invasion of BC cells with different BM abilities. Importantly, SNORA71B promoted the EMT process of low BM BC cells. SNORA71B knockdown inhibited the high BM BC cells across BBB, while EMT activator dramatically abrogated this inhibited effect.

**Conclusions:**

In conclusion, SNORA71B promotes BC cells across the BBB partly via inducing EMT.

## Introduction

Breast cancer (BC) is the most common cancer in women patients and develops to brain metastases (BM), which further severely impairs patients’ quality of life. The majority of BM mainly occur in those with lung, breast, and colorectal cancers, melanoma or renal cell carcinoma. Brain metastasis of breast cancer accounts for 5–20% among those cancer [1]. However, women patients with breast carcinoma have higher incidence of brain metastases compared with men patients [2]. Moreover, the median survival time of breast cancer brain metastases (BCBM) patients is between 4 and 6 months [3]. Genes related with cell growth, proliferation, and tumor suppressors participate in BCBM process, regulated by transcription, post-transcription level, and epigenetic modification [4]. BM develop following the spread of cells from a primary tumor to the brain microvasculature through the blood [5]. Moreover, a new metastasis (colonization) is caused by micro-environmental niche–tumor interactions, neuroinflammatory cascades, and neovascularization [5]. Although current therapeutic strategies are used for BCBM patients including whole-brain radiation therapy (WBRT), surgical resection, and targeted therapies, BCBM has a dismal prognosis after the treatment [1, 6]. Moreover, options of effective targeted treatment are still finite for BCBM patients [7]. Although penetration of the blood–brain barrier (BBB) is one of the breakthroughs for effective therapy of BM, the effective treating substances for BM and the regulation molecules that inhibit tumor cells crossing the BBB is still unknown [8]. Moreover, the underlying mechanism of BC cells across the BBB is still explored.

SnoRNAs are a class of small non-coding RNA molecules (60–300 nt) widely presenting in the nucleolus of eukaryotic cell [9]. The biological processes of snoRNAs involvement are mainly processing of rRNA, regulation of RNA alternative splicing, and translation processes as well as oxidative stress response [10]. With the development of second-generation sequencing technology, more data indicate that snoRNAs are abnormally regulated in tumors and also involved in hereditary diseases, hematopoiesis, metabolism, and cancer [11, 12]. SnoRNAs were recently reported to play important biological function in other types of cancer. The significance as oncogenic signal modulators has attracted increasing attention. For instance, SNORD43, SNORD44, and SNORD48 act as potential inhibitors of BC, head, and neck squamous cell carcinoma [13]. SNORD113-1 is down-regulated and functions as a suppressor gene in primary hepatocellular carcinoma [14]. Several snoRNAs located in frequently amplified genomic regions are up-regulated in non-small cell lung cancer [15, 16]. The new molecular biomarkers have been identified between BCBM and both BC and primary brain tumors (prBT), including the representative molecules (SNORA71A, SNORA71B, and SNORA71C) [17]. However, the role of snoRNAs participating BCBM has not been investigated still.

In this study, to investigate the potential function of SNORA71B associated with BC metastasis to brain, we utilized GEO and SNORic databases to analyze the difference expression (DE) of snoRNAs in normal tissues, low and high BM BC tissues, and verified DE SNORA71B by qRT-PCR. We assessed the effect of SNORA71B on the abilities of proliferation, migration invasion, and EMT process as well as BC cells across the BBB.

## Materials and methods

### Analysis of snoRNA from GEO and SNORic databases

We queried Gene Expression Omnibus (GEO) database (www.ncbi.nlm.nih.gov/geo/) to download microarray data under accession number GSE100534, including 19 cases of BC tissue samples and 3 cases of BCBM tissue samples data. Further, the data was analyzed to screen DE snoRNAs with high fold change value. Additionally, the snoRNAs in breast cancer tissues and normal tissues were investigated based on snoRNA sequencing data. We downloaded snoRNA expression levels from at http://bioinfo.life.hust.edu.cn/SNORic/download, which provides 4 modules, including summary, snoRNA-based analysis, gene-based snoRNAs, and download. Moreover, the correlation between snoRNAs and CNV, DNA methylation, mRNAs, RNA splicing events, and protein expression can be examined. Based on the SNORic database, 1077 patients with breast cancer tissues and 105 normal tissues were analyzed. In addition, there were 103 matching pairs. We mapped the reads to snoRNA genes and quantified the expression of snoRNAs as RPKM. snoRNAs with an average RPKM ≥ 1 in each sample were defined as detectable snoRNAs. A paired Student’s t test was used to assess the statistical difference in the snoRNA expression level between tumor and normal samples. The differentially expressed snoRNAs met the following criteria: adjusted p value  <  0.05 and an absolute log fold change >  1.00.

### Kaplan–Meier survival curve analysis

The prognostic values of screened snoRNA in BC were analyzed via the Kaplan–Meier plots (http://kmplot.com/analysis/), which is an online database containing gene expression profiles and survival data of BC patients. According to the expression of screened snoRNA, the cases in the database were ranked from low expression to high expression. The bottom 50% were divided into the low expression group and the top 50% belonged to the high expression group. All cohorts were compared with KM survival plots. Hazard ratio (HR), 95% confidence interval (95% CI), and log rank *P* value were calculated.

### Cell culture

High BM BC cell lines (MDA-MB-231BM), low BM BC cell lines (MDA-MB-231, SKBRS, MDA-MB-468, MCF-7), normal BC cell lines (MCF10A), human brain microvascular endothelial cell (HBMEC), and human astrocytoma cell (HAC) were obtained from the American Type Culture Collection (Manassas, VA, USA). With the exception of HBMEC cultured in endothelial cell medium (ECM) (ScienCell Research Laboratories, CA, USA), the other cell lines were maintained in DMEM medium (Corning, NY, USA) containing 10% FBS (FBS, ScienCell, USA) plus 100 U/mL penicillin/streptomycin at 37 °C in a 5% CO2 of humid environment.

### Reverse transcription and real-time PCR

Total RNA of BCBM, BC, and normal breast cells was extracted by Trizol reagent (Invitrogen, Carlsbad, CA, USA). The integrity of RNA was assessed using agarose electrophoresis. RNA concentration was measured by Nanodrop 2000 (Tiangen, Beijing, China). Then the total RNA was reverse transcribed to cDNA using the RETROscript kit (Life Technologies, Rockville, MD, USA). The primers for SNORA71BD were designed by Primer Premier 6 and the level of SNORA71B expression was measured with SYBR Green Master Mix using StepOnePlusTM Real-Time PCR System (Applied Biosystems). GAPDH was used as endogenous control, and the fold change was calculated via the 2-∆∆Ct method. Each experiment was performed three times. The primer sequences are as shown in Table 1.

**Table 1:** Primer sequences

Primer name	Sequence (5′–3′)
GAPDH-F	AGAAGGCTGGGGCTCATT
GAPDH-R	TGCTAAGCAGTTGGTGGTG
SNORA71BD -F	GAGAGGAATCAATGAAAGCGCT
SNORA71BD -R	GCATGTACGAAAGCTCCAGAGTT
NC-F	UUCUCCGAACGUGUCACGUTT
NC-R	ACGUGACACGUUCGGAGAATT
siSNORA71B-26-F	UGCCUUUGCCCUGGUCAUUTT
siSNORA71B-26-R	AAUGACCAGGGCAAAGGCATT
siSNORA71B-96-F	CCACUCCUAUCCCUUCCAATT
siSNORA71B-96-R	UUGGAAGGGAUAGGAGUGGTT

### RNA interference assay

Small interfering RNAs of SNORA71B and the negative control siRNA were purchased from Shanghai GenePharma Company. The siSNORA71B were transfected into different BM BC cells with Lipofectamine 2000 (Invitrogen, Carlsbad, CA, USA). qRT-PCR was performed to detect and screen interference efficiency of siSNORA71B for subsequent functional experiments. The primer sequences of small interfering RNAs are as shown in Table 1.

### Plasmid construction and transfection

The synthesized SNORA71B was cloned into pcDNA3.1 vector (Life Technology). The constructs were confirmed by sequencing and termed as p3.1-SNORA71B. The expression of SNORA71B was verified by qRT-PCR. The packaging plasmid pcDNA3.1-SNORA71B and vector pcDNA3.1 were transfected into low BM BC cell lines (MDA-MB-231) with Lipofectamine 2000, respectively; after transfection for 48 h, cells at 80–90% confluence were collected. Then, the levels of SNORA71B over-expression were detected.

### Cell counting kit-8(CCK8) assay

The different BM BC cells (2 × 10^3^ cells/well) were seeded into 96-well plates for CCK8 assay and cultured at 37 °C in 5% CO_2_, and siSNORA71B and siNC were transfected into the high BM BC cells with Lipofectamine 2000, respectively. In addition, pcDNA3.1-SNORA71B and pcDNA3.1 were, respectively, transiently transfected into the low BM BC cells. After 2 days, the cells were incubated with 10 μl of CCK8 reagent (Beyotime, Shanghai, China) at 37 °C for 4 h at the indicated time point for 0 h, 24 h, 48 h, 72 h, and 96 h. The absorbance at 450 nm was measured with Infinite M1000 instrument (Tecan Austria GmbH, Grödig, Austria). Each experiment was performed in triplicate.

### Migration and invasion assays

The cell invasion assays using BioCoat™ Matrigel^®^ Invasion Chamber (8 μm, pore size, biocoat) were conducted according to instructions. Briefly, the different BM BC cells (5 × 10^4^ cells/well) were plated with 500 μl DMEM into the upper chamber. DMEM (700 μl) supplemented 10% FBS was added in the bottom chamber. After the different BM BC knockdown or over-expression for 24 h, the cells were fixed with methanol (800 μl) and stained with crystal violet (Beyotime, Shanghai, China) for 30 min. Cells that did pass through the pores were removed with a cotton swab. The numbers of cells were counted on the lower surface of the membrane. The basic steps of cell migration assays were the same as above, except the difference of transwell chamber not coated with Matrigel. Each experiment was performed in triplicate.

### Western blot analysis

Total different BM BC cell lysates were performed. Protein lysate samples were separated by preparing 4% stacking gel and 10% separating gel, and then transferred onto PVDF membranes. The membranes were blocked in TBST in 5% fat-free milk for 1 h at room temperature. Next, the PVDF membrane was incubated with special primary antibodies: Vimentin (Abcam, Cambridge, UK; 1:500) overnight at 4 °C, E-cadherin (Santa Clara, CA, USA; 1:500), GAPDH (Proteintech, Chicago, UK; 1:1000). The membrane was washed three times for 1% TBST and subsequently incubated for 1 h with related goat anti-mouse IgG-HRP secondary antibodies (Beyotime, Shanghai, China). Then, protein terms were visualized using Novex™ ECL Chemiluminescent Substrate Reagent (Invitrogen, Carlsbad, CA, USA).

### Transwell invasion assay using the in vitro BBB model

An in vitro BBB model method referred in the report was utilized to estimate invasion ability of BCBM cells (Tominaga N et al. 2015). The in vitro BBB model consisted of HAC (2 × 10^4^ cells/well) seeded on the back face of BioCoat™Matrigel^®^ Invasion Chamber (8 μm pore size, biocoat) and HBMEC laid directly in the upper chamber (2 × 10^4^ cells/well). After appropriate time point, the back face and the front face of invasion Chamber had formed. Culture medium was, respectively, removed from the upper chamber. Then high BM BC cells (5 × 10^4^ cells/well) planted separately in the upper chamber, and further were transfected with siSNORA71B and NC. After 24 h, the invasive cells across the cell barrier and marked FITC on the lower Chamber were photographed under through fluorescence microscope (20 ×) (Nikon, TE300) and quantified by counting in five randomly selected areas.

### Statistical analysis

Survival curves were analyzed by the Kaplan–Meier plots. For statistical analysis, data from three independent experiments are presented as the mean ± S.D. The different gene expression levels of SNORA71B in tumor and normal samples were analyzed using two-way sample *t* test.* P* values were calculated by Graph prism8, using Student’s t test in two group comparison or one-way analysis of variance (ANOVA) followed by Tukey’s multiple comparison test in more than two groups. Only *P* < 0.05 was considered to be statistically significant.

## Results

### SNORA71B is up-regulated in relative higher BM BC cells

**Fig. 1:**
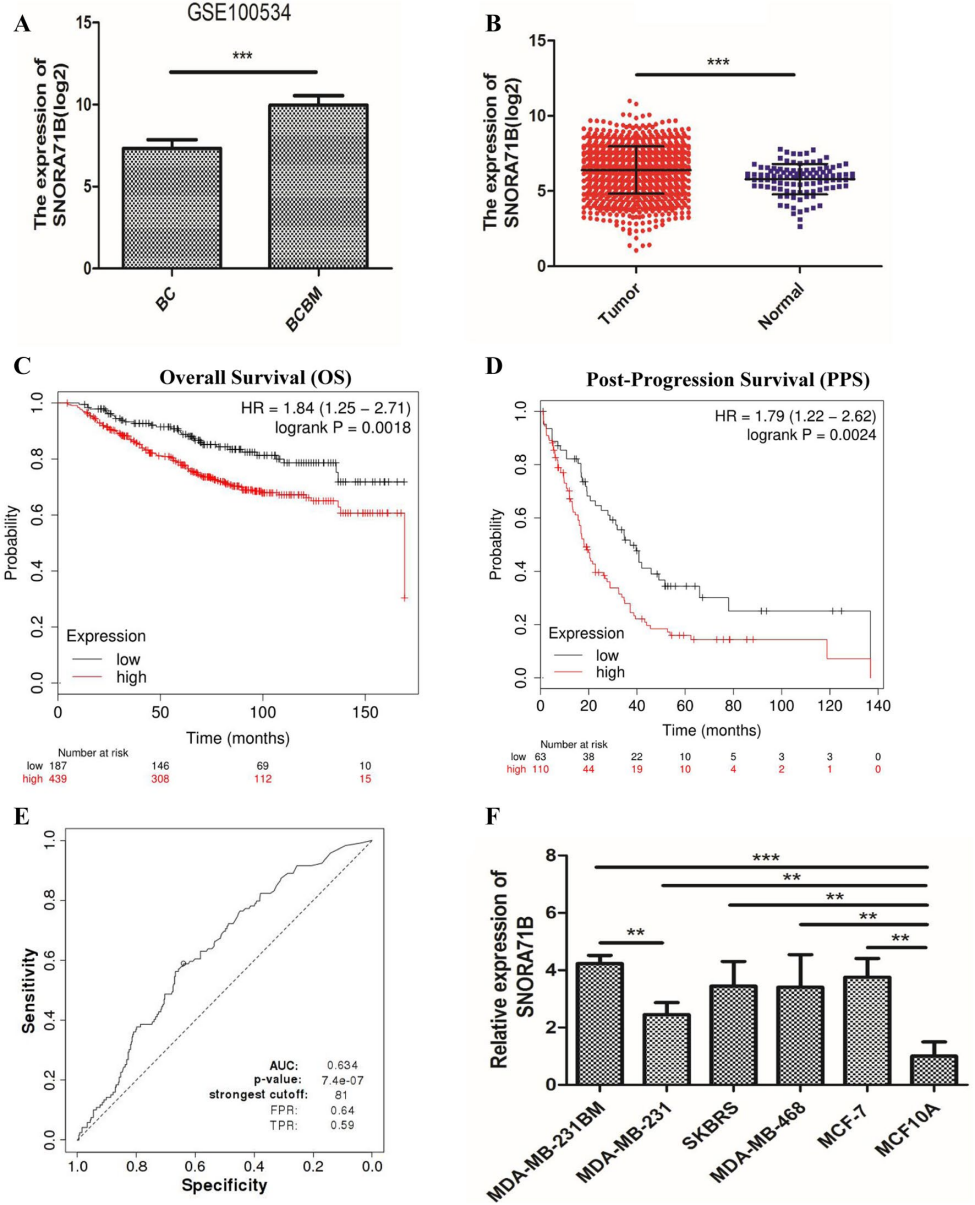
The expression of SNORA71B was higher in relative high brain metastatic BC tissues and cells, and Kaplan–Meier survival curve analysis of the prognostic significance of high and low expression of that in breast cancer. **a** SNORA71B expression was high in BCBM tissues compared with BC tissues through GSE100534 datasets analysis. **b** SNORA71B expression was high in breast tumor tissues compared with normal breast tissues via SNORic datasets analysis. **c**, **d** Low SNORA71B expression was correlated with better Overall Survival (OS) and Post-Progression Survival (PPS) in breast cancer (*n* = 626, *n* = 173). **e** The receiver operating characteristics (ROC) curves of SNORA71B in breast cancer patients. Notes: The area under the ROC curve was 0.634, (*P* value = 7.4e-07). **f** The expression level of SNORA71B in the high (MDA-MB-231BM) and low (MDA-MB-231, SKBRS, MDA-MB-468, and MCF-7) BM BC cells and normal breast cells (MCF10A) using qRT-PCR analysis. The data represent three repeated experiments, presented as mean ± SD. **P < 0.05, **P < 0.01, ***P < 0.001

To screen snoRNAs related to BC metastasis, we first loaded GSE100534 microarray data and found that the expression of SNORA71B was significantly different and higher in BCBM tissues compared with relative lower BM BC tissues (Fig. 1a). According to the SNORic data analysis, the expression of SNORA71B was high in breast tumor tissues compared with normal tissues (Fig. 1b). Additionally, the Post-Progression Survival (PPS) and Overall Survival (OS) using Kaplan–Meier Plotter (http://kmplot.com/analysis/) analysis results showed that low expression of SNORA71B was correlated with better Overall Survival (OS) and Post-Progression Survival (PPS) in breast cancer patients (Fig. 1c, d). We also analyzed the diagnosis ability of SNORA71B via receiver operating characteristic (ROC) curve analysis. The result showed SNORA71B might act as biomarker for breast cancer, with the area under curve (AUC) of 0.634 (*P* value = 7.4e-07) (Fig. 1e). At the cutoff value, the sensitivity and specificity of breast cancer diagnosis were 64% and 59%, respectively. Subsequently, the levels of SNORA71B expression were also verified in different metastatic ability BC cells. The result revealed that the expression of SNORA71B was up-regulated in high BM BC cells (MDA-MB-231BM) compared with relative lower BM BC cells (MDA-MB-231, SKBRS, MDA-MB-468, MCF-7, and MCF10A) (Fig. 1f).

### SNORA71B promoted proliferation of different metastatic ability BC cells

**Fig. 2:**
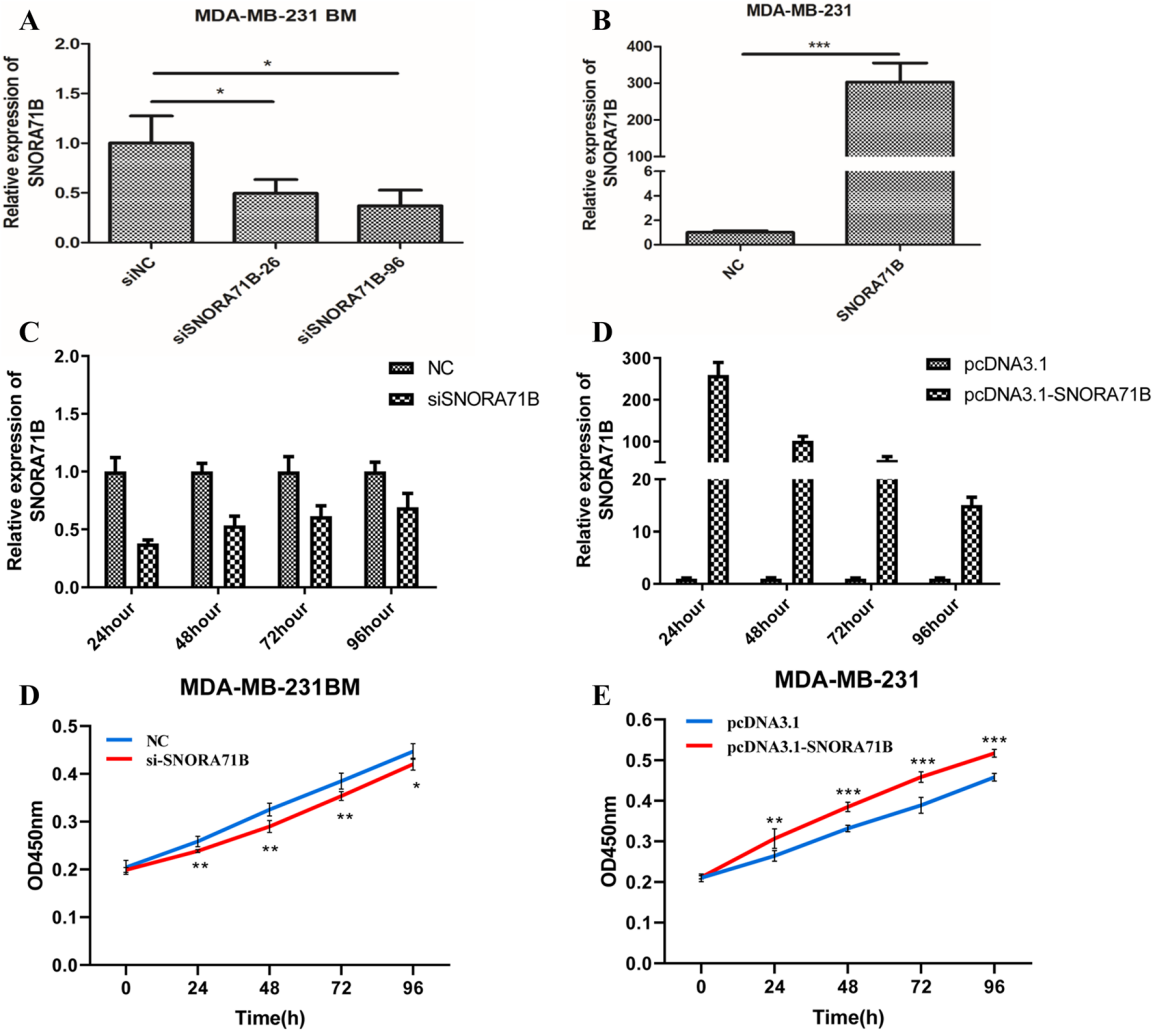
The SNORA71B promoted proliferation of different metastatic capacity BC cells. **a** qRT-PCR analysis of relative SNORA71B expression in siSNORA71B-infected high BM BC cells (MDA-MB-231BM). **b** The relative expression of pcDNA3.1-SNORA71B in SNORA71B-transfected low BM BC cells (MDA-MB-231) using qRT-PCR assay. **c**, **d** qRT-PCR detected expression stability of SNORA71B expression at 24 h, 48 h, 72 h, and 96 h after pcDNA3.1-SNORA71B and siSNORA71B transfection. **e, f** CCK8 assays were utilized to evaluate effect of SNORA71B on the proliferation abilities of the high and low BM BC cells (MDA-MB-231BM, MDA-MB-231). Values are indicated as mean ± SD from three independent experiments. *P < 0.05, **P < 0.01

To assess whether SNORA71B play a functional role in different metastatic BC cells, we first detected the effect of SNORA71B on cell ability of proliferation in vitro. The results showed the levels of SNORA71B were decreased after knocking down SNORA71B in MDA-MB-231BM cells (Fig. 2a). Moreover, the levels of SNORA71B were increased after SNORA71B over-expression in the MDA-MB-231 cells (Fig. 2b). Additionally, after transient transfection, the stability of siSNORA71B and pcDNA3.1-SNORA71B was assessed during 96 h by qRT-PCR. The SNORA71B expression of MDA-MB-231BM cells transfected with siSNORA71B decreased at 24 h, 48 h, 72 h, and 96 h (Fig. 2c). After transfecting pcDNA3.1-SNORA71B, the SNORA71B expression of MDA-MB-231 cells increased at 24 h, 48 h, 72 h, and 96 h (Fig. 2d). After SNORA71B knockdown, the proliferation ability of MDA-MB-231BM cells at 48 h, 72 h, and 96 h was impeded compared with NC group, respectively (Fig. 2e). Consistent with the increase of SNORA71B level, the proliferation ability of MDA-MB-231 cells at 48 h, 72 h, and 96 h was promoted compared with pcDNA3.1 group, respectively (Fig. 2f).

### SNORA71B promoted migration of different metastatic ability BC cells

**Fig. 3:**
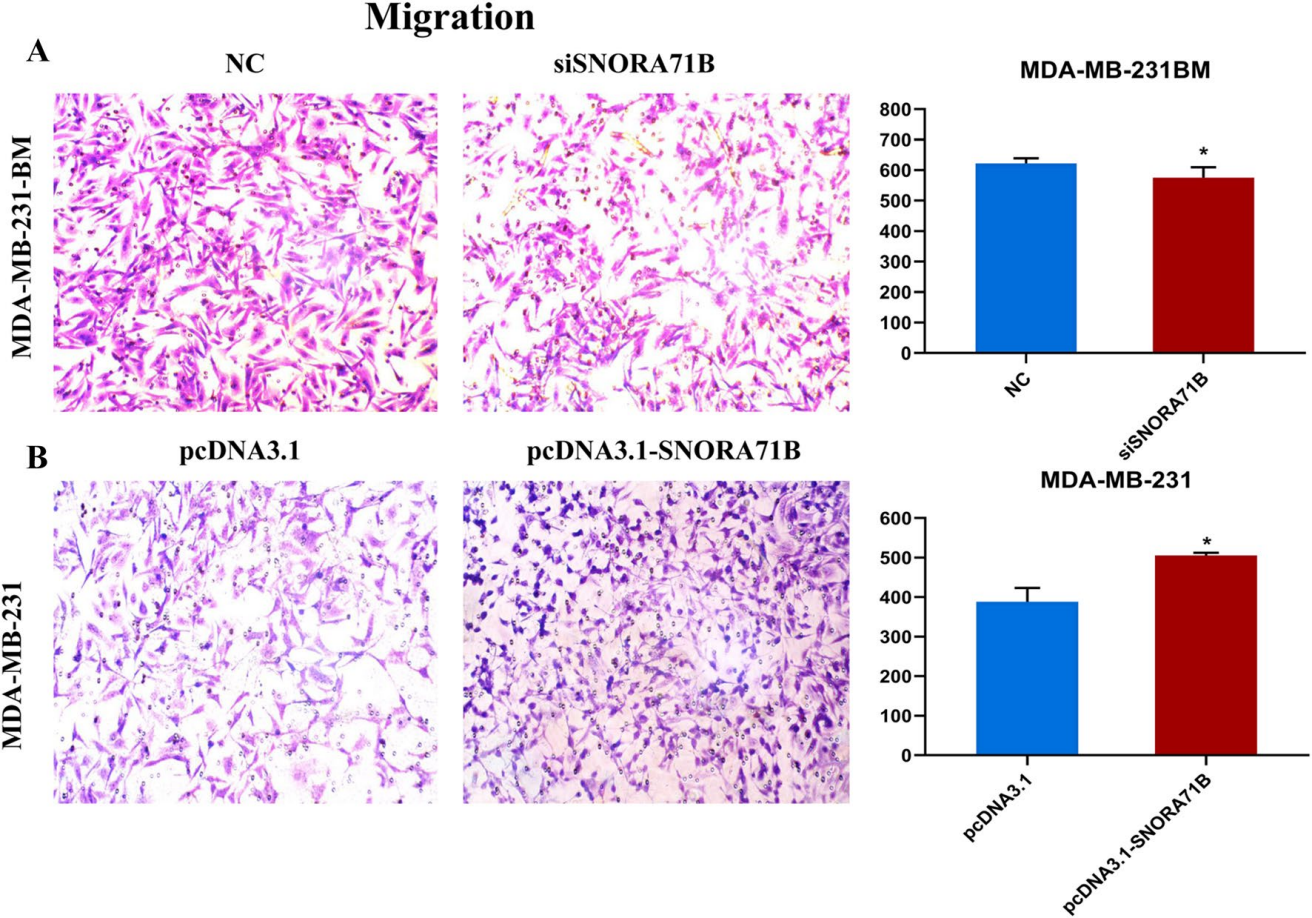
The SNORA71B promoted migration in different metastatic capacity BC cells. **a**, **b** Migration abilities of high BM BC cells (MDA-MB-231BM) infected with siSNORA71Bs and low BM BC cells (MDA-MB-231) transfected with pcDNA3.1-SNORA71B were analyzed by chamber migration assays. The results are presented as the mean ± standard error of the mean (*n* = 3). *P < 0.05

We further examined whether SNORA71B affected migration capacity of different metastatic ability BC cells. It is revealed that SNORA71B knockdown repressed the migration capacities of the high BM BC cells (Fig. 3a). Moreover, the up-regulation of SNORA71B promoted migration ability in the low BM BC cells (Fig. 3b).

### SNORA71B promoted invasion of different metastatic capacity BC cells

**Fig. 4:**
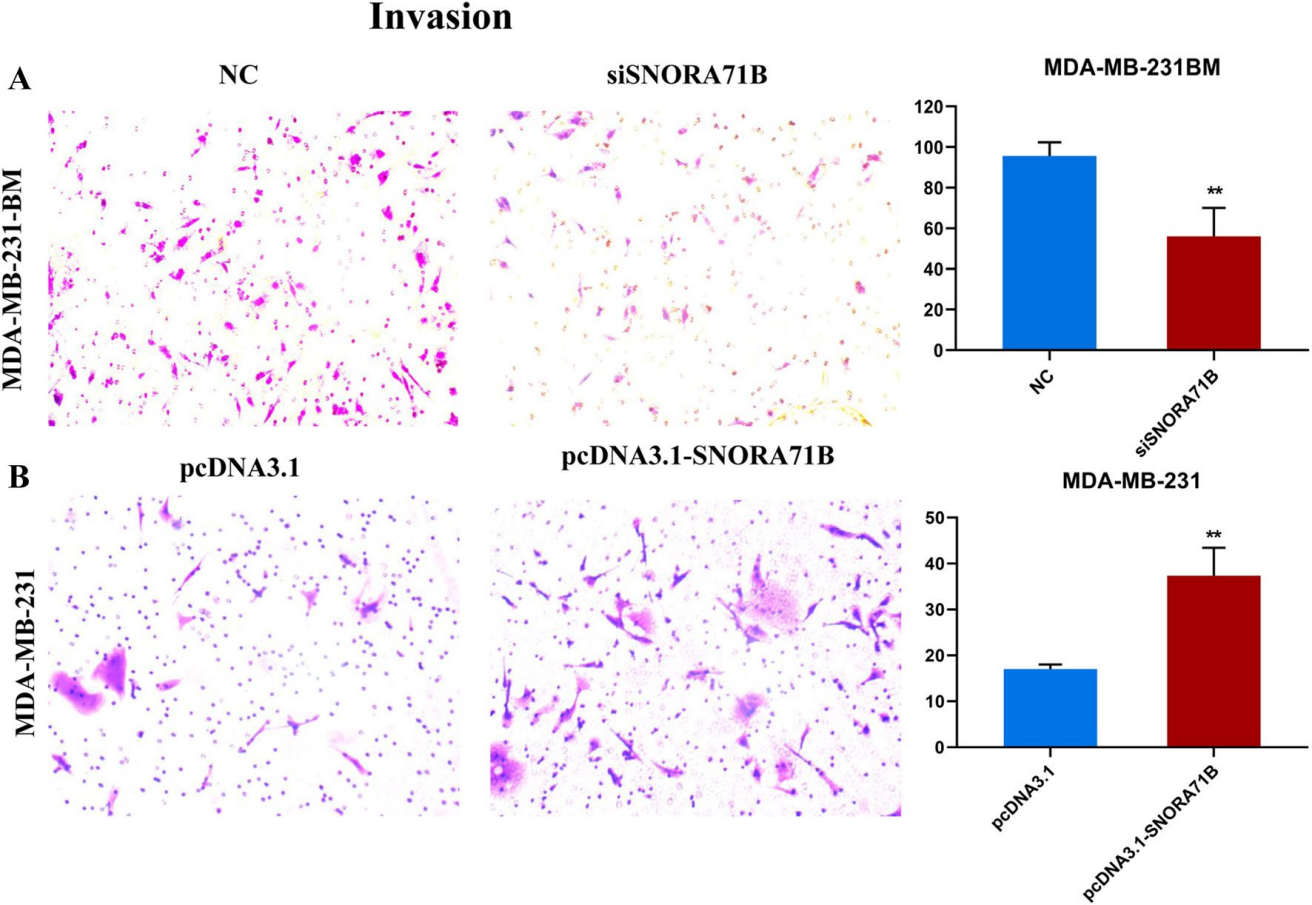
The SNORA71B promotes invasion of different metastatic capacity BC cells. **a**, **b** Invasion abilities of high BM BC cells (MDA-MB-231BM) infected with siSNORA71Bs and low BM BC cells (MDA-MB-231) transfected with pcDNA3.1-SNORA71B were analyzed by chamber invasive assays. Data are the means ± SDs (*n* = 3). *P < 0.05; **P < 0.01

Subsequently, we explored whether SNORA71B affected the invasion capacity of different BM BC cells. The result indicated that the invasion abilities of high BM BC cells infected with siSNORA71Bs were inhibited (Fig. 4a). Meantime, the over-expression of SNORA71B promoted invasion in low BM BC cells (Fig. 4b).

### SNORA71B advanced EMT progress, and silencing of SNORA71B inhibited the high BM BC cells across BBB in vitro

**Fig. 5:**
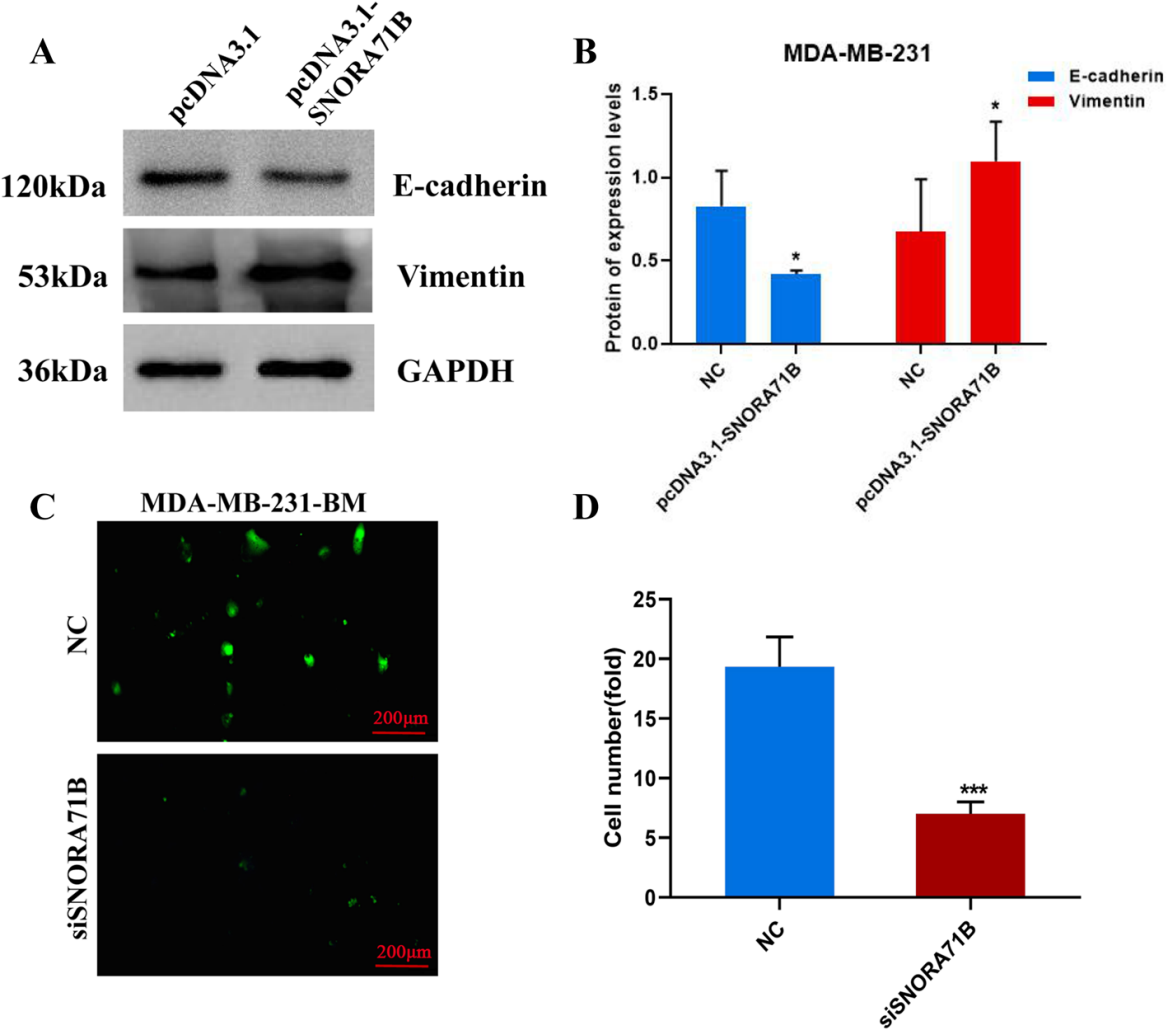
SNORA71B promotes breast cancer cell epithelial-mesenchymal transition and across blood–brain barrier. **a**, **b** Western blot assay was exploited to analyze the effect of SNORA71B on EMT progress in SNORA71B-transfected low BM BC cells (MDA-MB-231). **c**, **d** Transwell invasion assay using the in vitro BBB model detected the effect of SNORA71B on high BM BC cells (MDA-MB-231BM) across the BBB. The results were presented as the means ± SDs (*n* = 3). *P < 0.05, **P < 0.01

The EMT of BC cells was the important event of BCBM. Therefore, we performed western blotting assay to examine levels of marker molecules about EMT. The result showed that the level of epithelial marker (E-cadherin) was significantly decreased, and mesenchymal marker (Vimentin) was increased in SNORA71B-transfected low BM BC cells (Fig. 5a, b). The SNORA71B promoted EMT progress, resulting in BC cell transition and spreading. In addition, the important process of brain metastasis was tumor cells across the BBB. To study whether the SNORA71B affected high metastatic breast cancer cells across the BBB, we established an in vitro BBB culture system. Fluorescence representative figures showed SNORA71B knockdown exhibited lower green fluorescence in high BM BC cells, indicating that fewer high BM BC cells passed the BBB model (Fig. 5c, d). Therefore, the SNORA71B promoted the high BM BC cells across BBB in vitro.

### SNORA71B promoted the high BM BC cells across BBB by EMT

**Fig. 6:**
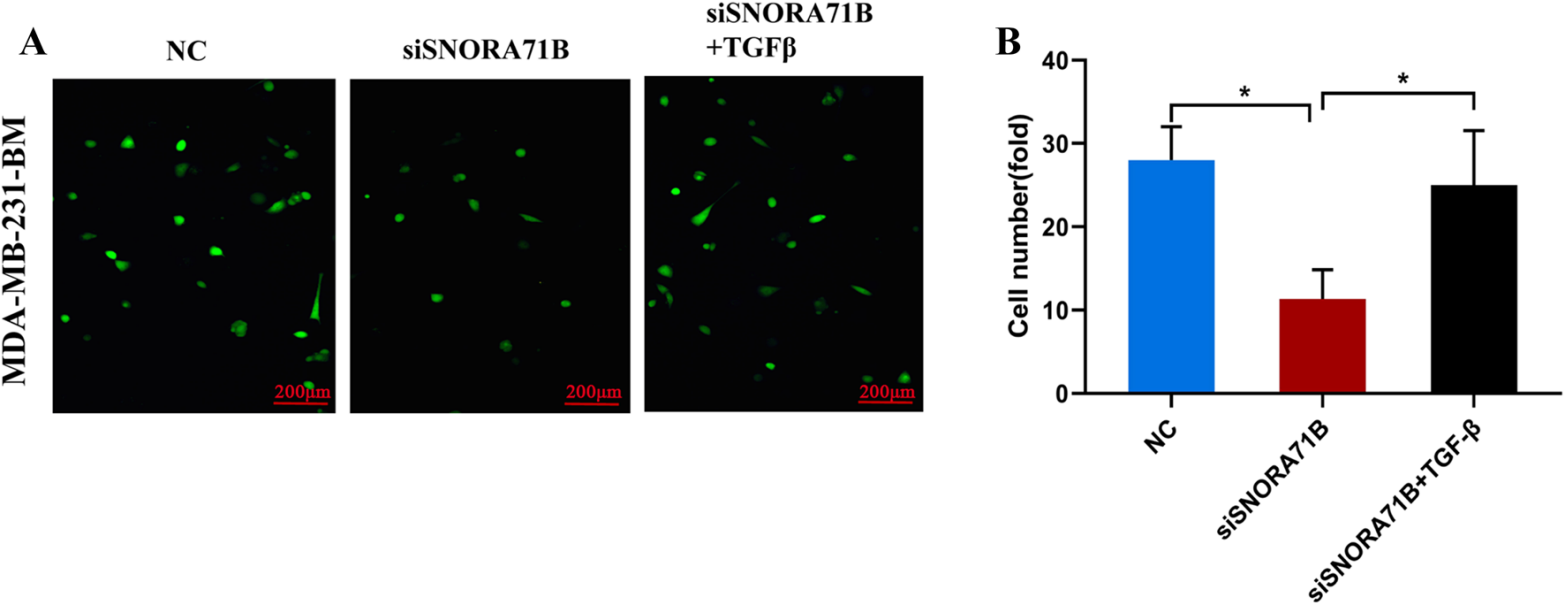
SNORA71B promotes breast cancer cells across blood–brain barrier via mediating epithelial-mesenchymal transition. **a d**, **e**. The effect of SNORA71B on high BM BC cells (MDA-MB-231BM) across the BBB via mediating epithelial-mesenchymal transition (EMT). Notes: TGFβ can mediate the entire EMT. The results were presented as the means ± SDs (*n* = 3). *P < 0.05

To study whether the SNORA71B mediated the BM BC cells across BBB by EMT, the in vitro BBB culture system was used. Moreover, the early reporters show TGFβ can mediate the entire switch from epithelial to mesenchymal phenotype (Miettinen and Ebner 1994). SNORA71B knockdown inhibited the high BM BC cells across BBB, while treatment with the TGFβ reagent promoted this decreased ability of the high BM BC cells across BBB (Fig. 6a, b).

## Discussion

The molecular basis and mechanisms of breast tumor metastasis are largely unknown [18]. The early reporters show a total 10% of patients with BC develop to brain metastasis disease [19]. With the improvement of diagnosis and treatment of BC, the survival period of BC patients is significantly prolonged. Moreover, the incidence of BC metastasis to brain is gradually increasing [20]. Presently, the patients with BCBM urgently need novel molecular biomarkers for the diagnosis and prognosis. Interestingly, we found that the level of SNORA71B is significantly higher in relative higher brain metastatic BC cells, indicating SNORA71B may be a regulator governing invasion and metastasis of breast cancer cells. Moreover, low expression of SNORA71B showed better PPS and OS in breast cancer patients, suggesting SNORA71B was correlated with unfavorable prognosis. SNORA71B might act as biomarker for breast cancer through ROC curve analysis. Together, SNORA71B might be a novel biomarker for the diagnosis and prognosis target in limiting the malignant progress of BC.

The role of snoRNAs acts as a cell steward until the discovery of snoRNAs participating human cancer. Emerging evidence showed that the functioning role of snoRNAs might be cell behavior control, and loss function of snoRNAs could remarkably contribute to tumorigenesis [21]. Presently, massive snoRNAs have been reported in various organisms. Two large families of snoRNAs nominated the two types of rRNA-modified nucleotides in eukaryotic, involved with the specific site formation [22]. Although multiple snoRNAs have been shown to play a vital role in tumors, most of snoRNA have not been characterized. SNORA71B is the latest potential biomarker of BCBM, discovered by sequencing technology [17, 23]. We still poorly know whether SNORA71B is involved in the process of breast cancer metastasis to brain. Here, the function role of SNORA71B showed that SNORA71B enhanced cell proliferation, migration, and invasion of different metastatic ability BC cells, indicating SNORA71B plays important role in BC cells during metastasis.

Brain metastasis is a complicated process including EMT. Metastasis is often depicted as a multistage process, in which the migration and invasion ability and EMT of malignant cells enhances during the primary stage, and spread from the tumour of origin to colonize distant organs [24, 25]. The EMT enables cancerous epithelial cells to enter the mesenchymal state, entrusting cancer cells with migration and invasion. The primary tumor can migrate and colonize the distal organs and form secondary tumor metastasis [26, 27]. The expression of most important molecular markers (E-cadherin) of EMT obviously decreased, suggesting the adhesion ability is significantly reduced in BC cells, further promoting the metastasis progress of BC cells. Recently, as was reported brain tissue of the directional transfer has obvious relationship with EMT [28, 29]. LncRNAs regulated the EMT process in brain metastasis [30, 31]. However, whether the snoRNAs are involved in tumor metastasis mediating EMT progress has not been reported. In the study, SNORA71B promoted down-regulation of e-cadherin and up-regulation of vimentin expression in low BM BC cells, indicating SNORA71B drives BC cell metastasis through promoting EMT progress.

In the past decades, studies have illustrated circulating monocytes play an important role between the BBB and homeostatic perturbations in the brain [32], and BBB mainly consists of the endothelium and surrounding cells, and comes into being a nature barrier to resist harmful molecules or cells [33]. Conventional therapies for BCBM have no effect on the impermeability of the BBB and chemoresistance. Lnc-BM and JAK2 induce BCBMs through regulating signal transduction between the brain microenvironment and BC cells [34]. MALAT1 protects the BBB after stroke [35]. Mir-155 regulates key functions of the brain endothelial barrier during inflammation, and affects the pathogenesis of inflammatory diseases in the central nervous system through damaging the BBB [36]. SNHG12 (snoRNA) promotes metastasis and tumorigenesis through regulating miR-199a/b-5p in hepatocellular carcinoma [37]. However, whether the snoRNAs regulate BC cells to pass the BBB for resulting in brain metastasis remains unknown. We established the BBB model consisting of endothelium and human astrocytoma cells, contributing to investigate the effect of SNORA71B on the ability of BC cell across BBB, and found that SNORA71B promoted high BM BC cells across BBB by EMT, indicating SNORA71B plays an important role in brain metastasis.

## Conclusions

In conclusion, SNORA71B has high expression in relative to high BM breast cancer tissues and cells, and promoted different metastatic ability BC cells’ proliferation, migration, invasion, and even EMT progress as well as BC cells across the BBB by EMT. Our study implied that SNORA71B might mediate BC cell metastasis and act as a therapeutic target to treat BCBM in future. Additionally, the OS, PPS, and ROC curve analysis suggested the prognostic and diagnostic roles of SNORA71B expression in BC patients.

## Data Availability

The datasets used and/or analyzed during the current study are available from the corresponding author on reasonable request.
